# Preparation of Modified Polycarboxylate by Pyrrolidone for Using as a Dispersant in Cobalt Blue Nano-Pigment Slurry

**DOI:** 10.3390/molecules29163940

**Published:** 2024-08-21

**Authors:** Qianqian Tang, Rong Yang, Jinnuo Li, Mingsong Zhou, Dongjie Yang

**Affiliations:** 1 Henan Key Laboratory of Function-Oriented Porous Materials, College of Chemistry and Chemical Engineering, Luoyang Normal University, 6 Jiqing Road, Yibin District, Luoyang 471934, China; lhltqq1987@163.com (Q.T.); 17333740259@163.com (J.L.); 2 State Key Laboratory of Pulp and Paper Engineering, School of Chemistry and Chemical Engineering, South China University of Technology, 381 Wushan Road, Tianhe District, Guangzhou 510640, China; yr662934@163.com (R.Y.); cedjyang@scut.edu.cn (D.Y.)

**Keywords:** modified polycarboxylate by pyrrolidone, dispersant, cobalt blue nano-pigment slurry

## Abstract

In this paper, N-vinylpyrrolidone was copolymerized with acrylic acid and itaconic acid by free radical polymerization, and a series of polyacrylic acid-co-itaconic acid-co-N-vinylpyrrolidone (PAIN) dispersants with different pyrrolidone ligand contents were synthesized and characterized. Then, the cobalt blue nano-pigment slurry (20 wt%) was prepared through a water-based grinding method, and the optimum grinding technology was explored and determined as follows: PAIN2 as a dispersant, a dispersant dosage of 10 wt%, and a grinding time of 480 min. According to this optimum grinding technology, the prepared pigment slurry had a significantly decreased agglomeration, the *D_90_* of which was 82 nm, and separately increased to 130 nm and 150 nm after heat storage for 3 and 7 days, exhibiting excellent heat storage stability. Additionally, its TSI value was also the lowest (1.9%), indicating good dispersion stability. The QCM and adorption capacity measuring results showed PAIN2 had a larger adsorption capacity, and the formed adsorption layer had a higher rigidity and was not easy to fall off. This was caused by both the interaction of carboxyl groups and the pyrrolidone ligand (strong coordination interaction) in PAIN2 with cobalt blue. The XPS and FT–IR measurements further proved the above-mentioned adsorption mechanism.

## 1. Introduction

In recent years, the inkjet printing technology, as a new ceramic surface decoration technology, has attracted widespread attention, which is beneficial to realize the networking, high precision, multi-color, no breakage, personalization, and fashionization of ceramic decoration, so as to reduce the costs and improve the decorative quality as well as production efficiency of ceramic products [1,2,3]. The key to inkjet printing technology lies in the use of ceramic ink. At present, the ceramic inks used for inkjet printing are mainly oil-based inks, which are prepared by dispersing the inorganic pigments in organic solvent and have disadvantages such as high costs and being easy to agglomerate and settle. Moreover, the ink is highly toxic and would produce a large quantity of pollutants, including volatile organic compounds and black smoke, during the sintering molding process of ceramic embryos, bringing serious environmental pollution. Under the general trend of carbon dioxide peaking and carbon neutrality, the human health and environmental harm caused by oil-based ceramic ink greatly restrict the sustainable development of the ceramic industry. Using water as a dispersion medium to prepare green and environmentally friendly water-based ceramic inks has become an ideal solution [4,5,6,7].

However, in the water-based ink system, the ceramic pigment (generally inorganic pigment) has a large particle size and is easy to settle. Therefore, various methods, such as sol-gel [8,9,10,11], chemical coprecipitation [12,13], hydrothermal treatment [14,15,16] and polymer precursor [17,18], are used to prepare nano-pigment particles to improve the stability of ceramic slurry. But these technologies are hard to operate, and meanwhile, their productivity efficiency is low, making them impossible to achieve in industrial applications [4]. Therefore, preparing nano-pigment slurry through a physical grinding process has become a major concern [19,20,21]. Grinding the pigment particles to a diameter close to that of the colloidal particles (within about 200 nm) can enhance the non-directional Brownian motion of the pigment particles and reduce the settlement caused by gravity. However, the Brownian motion between nano-pigment particles is intense and the collision is frequent, which makes the dispersant easy to fall away from the particle surface, leading to the decline of the repulsion energy barrier among pigment particles. Therefore, the pigment particles are easy to agglomerate and settle [4,22,23]. In view of this, developing a water-based polymer dispersant with excellent adsorption properties to match the water-based ceramic pigment slurry is urgent.

Among various types of water-based dispersants, the polyelectrolyte dispersants represented by polycarboxylate are the most widely studied [6,24,25,26]. The key to polycarboxylate as a highly efficient dispersant for inorganic particles lies in the interaction between its carboxyl groups and inorganic particles, which is beneficial to the adsorption of polycarboxylate on the inorganic particles, thus enabling the particles to obtain good dispersion stability in aqueous solutions [27]. However, the effective adsorption capacity and wetting ability of polycarboxylate dispersants on the inorganic particle surface are not very good, which leads to an unsatisfactory grinding effect. Therefore, facilitating the effective adsorption of polycarboxylate on the ceramic pigment particles is the key to improving its performance. Qiao et al. [28] prepared a star-shaped polymer dispersant. In this dispersant, the sulfonate end acted as a hydrophilic group to stabilize it in the aqueous phase, and the hydrophobic polypropylene glycol main chain acted as an anchoring group to adsorb on the carbon nanotube surface. With the help of this star-shaped surfactant, the multi-walled carbon nanotube could be well dispersed in a high-concentration aqueous suspension for more than 30 days. But when sodium dodecyl benzene sulfonate was used as a dispersant, the carbon nanotube would completely separate from the suspension after 10 days. With the aim of stabilizing nanometer titanium dioxide (TiO_2_), Monteiro et al. [29] synthesized poly(ethylene glycol)-block-poly(4-vinyl pyridine) (mPEG-b-P4VP) amphiphilic block copolymers, and compared them with sodium polyacrylic acid (Na-PAA) dispersant, which was often utilized for TiO_2_ dispersion. The results showed that mPEG-b-P4VP had better performance than Na-PAA in maintaining dispersion stability and reducing particle size. North et al. [30] synthesized zwitterionic diblock copolymers containing cationic 2-(dimethylamino)ethyl methacrylate (DMA) and anionic methacrylic acid (MAA) repeat units through a one-pot method. It was found that PMAA, in the structure of this dispersant, can play an effective anchoring role. The addition of this dispersant can significantly improve the rheological properties of transparent yellow iron oxide pigment slurry as well as its dispersion stability. Based on the above research status, it is inferred that introducing anchoring groups onto the main chain of polycarboxylate may be a feasible solution to its weaker adsorption, which could make the dispersant maintain a strong single-point or multiple-point adsorption on the pigment particle surface to improve its adsorption strength. The pyrrolidone ligands in N-vinylpyrrolidone (NVP) can be used as anchoring groups. NVP is a water-soluble lactam compound and one of the few monomers with good biocompatibility. NVP has a five-membered ring containing N, and both O and N in its molecular structure have lone pairs of electrons, which can form coordination bonds with electron-poor acceptors such as most heavy metal ions [4,31,32].

In this paper, to solve the low adsorption strength problem of traditional polycarboxylate dispersants on the inorganic pigment particle surface, pyrrolidone ligands were introduced as anchoring adsorption groups to prepare modified polycarboxylate so as to enhance the adsorption performance of dispersants on the nano-pigment particles. To be specific, NVP was copolymerized with itaconic acid (IA) and acrylic acid (AA) at different molar ratios through free radical polymerization in aqueous solutions, and a series of polyacrylic acid-co-itaconic acid-co-N-vinylpyrrolidone (PAIN) dispersants with different pyrrolidone ligand contents, named PAIN1, PAIN2, and PAIN3, were synthesized, followed by being characterized by a Fourier transform infrared (FT–IR) spectrometer, ^1^H nuclear magnetic resonance (^1^H NMR) spectrometer, elemental analyzer (EA), and gel permeation chromatographer (GPC). Then, taking the commonly-used pigment cobalt blue (CoAl_2_O_4_) in ceramic inks, which had the advantages of stable chemical properties, bright colors, environmental protection, and no toxicity, as a research object and PAIN as the dispersant, the CoAl_2_O_4_ nano-pigment slurry was obtained by a water-based grinding method, and the optimum grinding conditions were explored. Subsequently, the performance of the CoAl_2_O_4_ nano-pigment slurry prepared by the optimum grinding technology, such as microstructure, particle size, heat storage stability, dispersion stability at room temperature, and XRD spectrogram, was determined. Next, the dispersion stability mechanism of PAIN on the CoAl_2_O_4_ nano-pigment slurry was investigated through zeta potential analyzer, quartz crystal microbalance (QCM), adsorption capacity, X-ray photoelectron spectroscopy (XPS), and FT–IR measurements, as well as the calculation of sedimentation displacement (*D*), diffusion displacement (*X*), and radius (*R_A_*) corresponding to the occupied area by each adsorbed PAIN molecule on the CoAl_2_O_4_ pigment particle surface. This work developed a new dispersant with excellent performance for the CoAl_2_O_4_ nano-pigment slurry as well as provided a theoretical reference for the structural design of functional polymer dispersants suitable for the water-based nano-inorganic pigment slurry system.

## 2. Results and Discussion

### 2.1. Characterizations of PAI and PAIN

**Figure 1:**
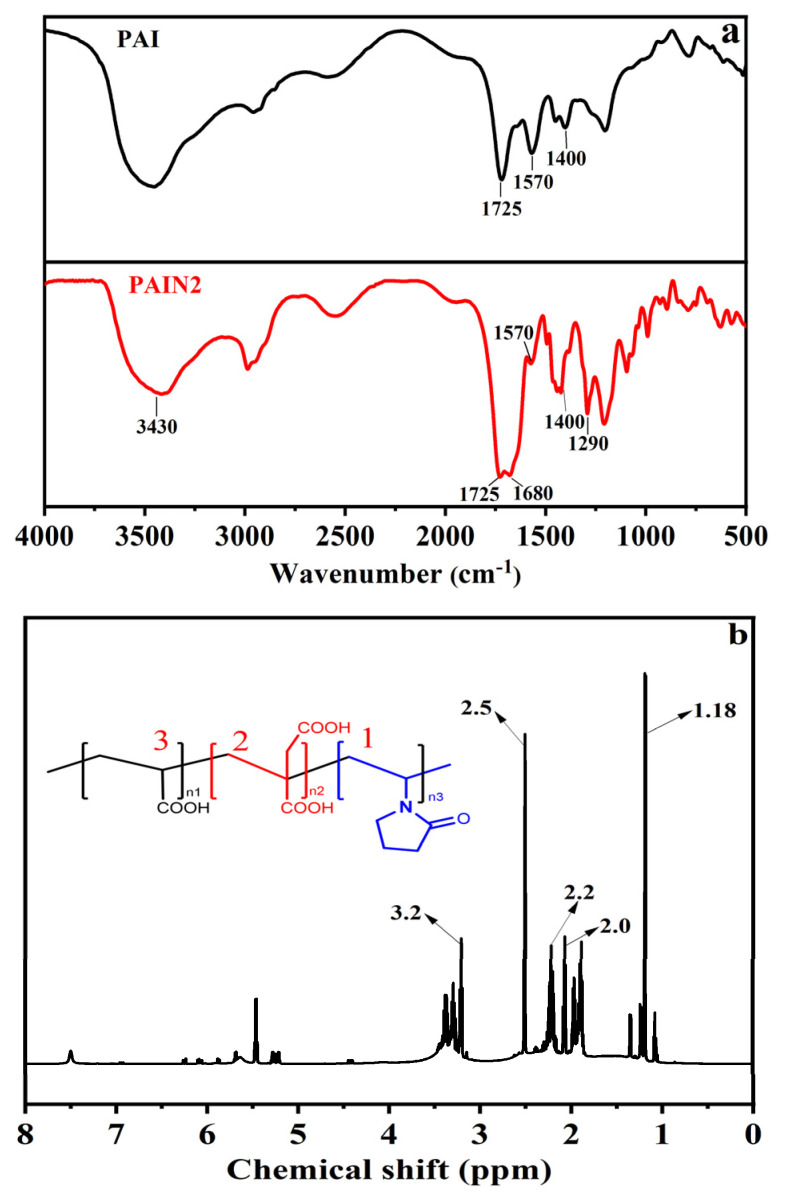
FT−IR spectra of PAI and PAIN2 (**a**), and ^1^H NMR spectra of PAIN2 (**b**).

Figure 1a gives the FT–IR spectra of PAI and PAIN2. As can be seen from Figure 1a, both PAI and PAIN2 showed signal peaks at 1725, 1570, and 1400 cm^−1^, which were all attributed to -COO^−^, indicating the presence of carboxyl groups in PAI and PAIN2. The peaks at 3430, 1680, and 1290 cm^−1^ in PAIN2 were separately caused by the stretching vibration of overlapping O-H and N-H, -C=O in the pyrrolidone ligand, and -C-N. This proved the successful synthesis of PAIN.

To further verify the structure of PAIN, a ^1^H NMR analysis was performed on PAIN2, just as illustrated in Figure 1b. The signal at the chemical shift of 2.5 ppm represented a solvent adsorption peak, and that at the chemical shift of 1.0–1.9 ppm was caused by the proton vibration of methylene away from the carboxyl group (−COOH) in the terpolymer (1). Due to the presence of two carboxyl groups, the methylene proton in polymerized IA in the trimer (2) moved to a low field, and its vibration peak appeared at about 2.0 ppm. Additionally, the signal at 2.2 and 3.6–3.0 ppm was attributed to the vibration of the proton in the methylene connected with −COOH (3) and pyrrolidone ligands, respectively.

The elemental analysis, pyrrolidone ligand content, and molecular weight data of PAI and PAIN are exhibited in Table 1. As more and more NVP was introduced into PAI to participate in the reaction, the nitrogen element content in the product PAIN gradually rose. By calculation, the pyrrolidone ligand content was found to increase from 1.89 mmol/L in PAIN1 to 3.16 mmol/L in PAIN2, and further to 3.38 mmol/L in PAIN3. However, the *M_w_* value of PAIN showed a decreasing tendency from PAIN1 to PAIN3. Overall, the synthesized three dispersants all had a relatively higher molecular weight and lower PDI. Using APS as an initiator can effectively control the preparation process of dispersants to obtain the desired product performance.

**Figure 2:**
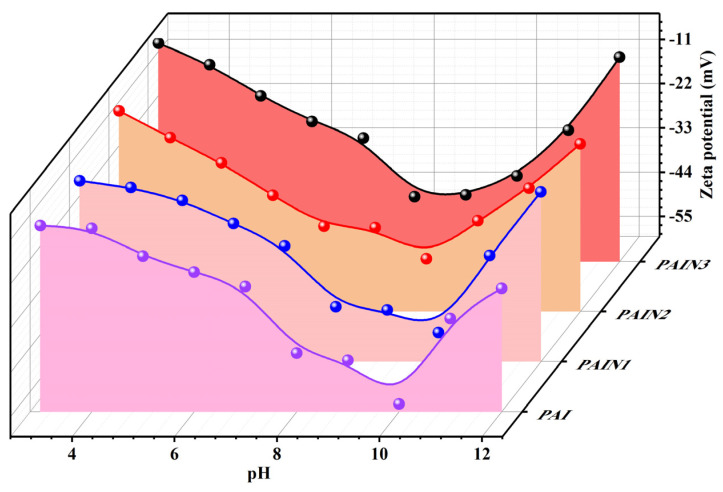
Zeta potential of PAI and PAIN aqueous solutions at different pH values.

Figure 2 shows the zeta potential of PAI and PAIN aqueous solutions at different pH values. It can be seen that all the systems were negatively charged, and the absolute zeta potential value of the synthesized PAIN products in aqueous solutions decreased with the increasing additive amount of NVP monomers. Besides, the pH value had an obvious impact on the zeta potential of dispersant solutions. Within the pH range of 5–11, the absolute zeta potential value of PAI and PAIN was both greater than 30 mV, indicating a relatively higher charge density. Especially at the pH value between 9 and 10, PAI, PAIN1, PAIN2, and PAIN3 all reached the highest absolute zeta potential values of 58.08, 52.79, 46.90, and 43.44 mV, respectively. Therefore, the addition of PAIN dispersant had the potential to improve the surface charge density of pigment particles.

### 2.2. Determination of the Optimum Wet Grinding Condition for Preparing CoAl_2_O_4_ Nano-Pigment Slurry

**Figure 3:**
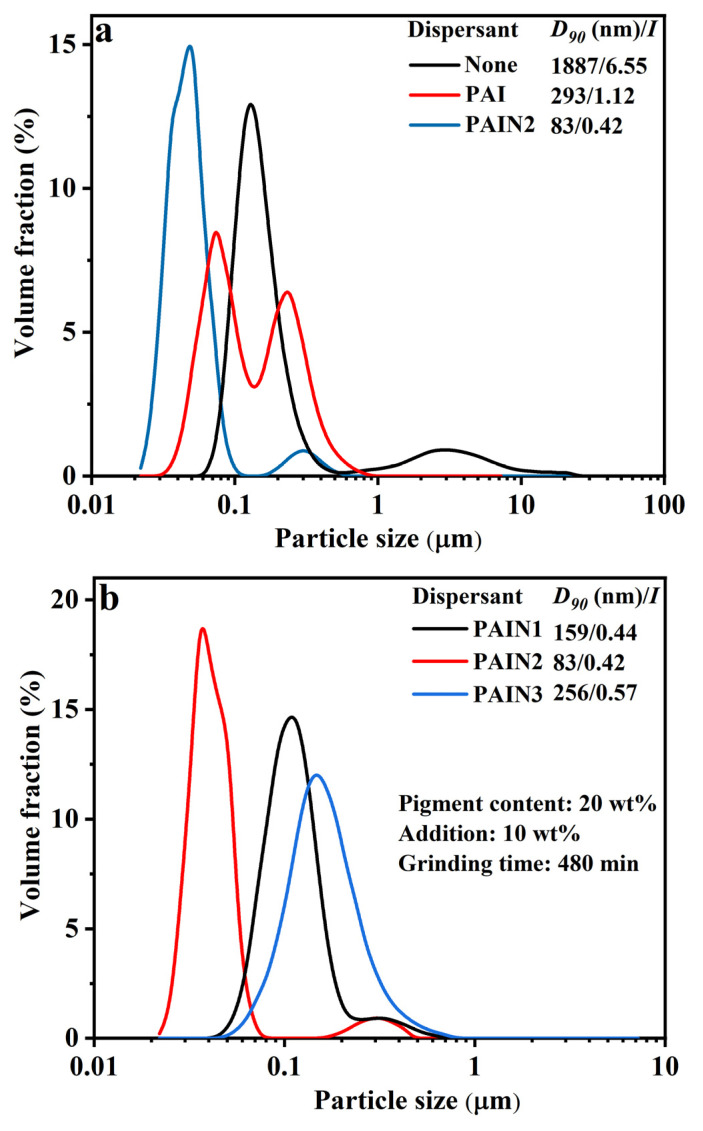

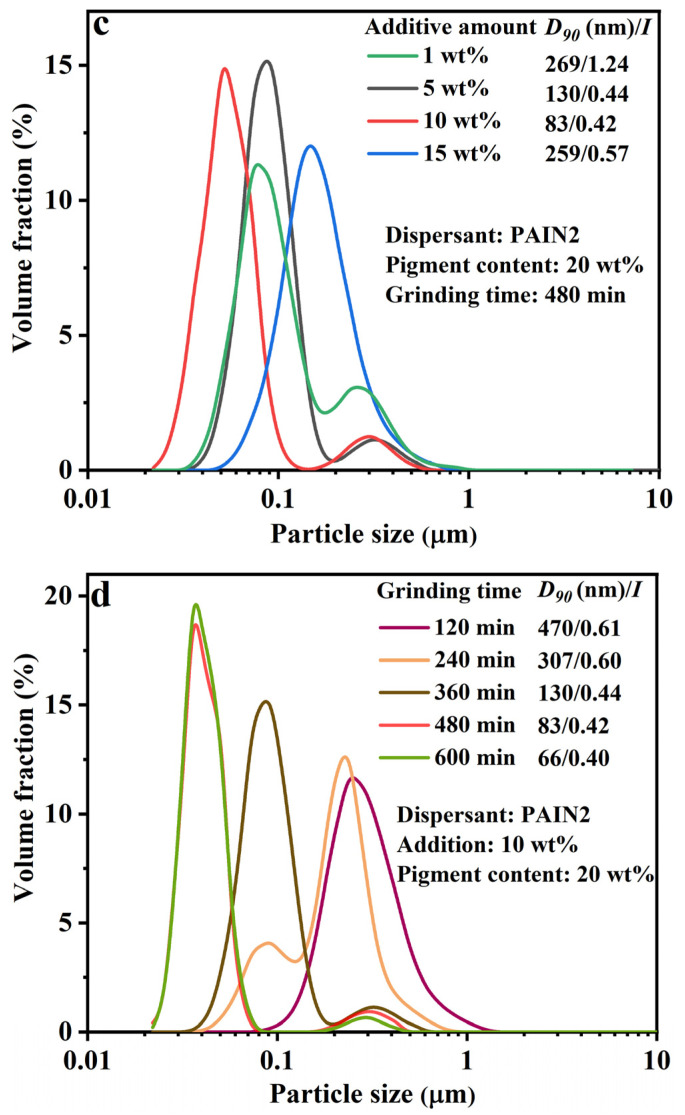
Effects of dispersant types (**a**,**b**), additive amount of dispersant (**c**), and grinding time (**d**) on the particle size distribution, *D_90_*_,_ and *I* values of CoAl_2_O_4_ nano-pigment slurry.

Firstly, the effect of dispersant types was studied. During the wet grinding process, the performance of the dispersant determined the particle size and particle size distribution of the pigment slurry. In this section, fixing the grinding time at 480 min and the CoAl_2_O_4_ pigment content at 20 wt%, the effects of adding 10 wt% of PAI or PAIN2 dispersant (or without any dispersant) to the *D_90_* of the pigment slurry are displayed in Figure 3a. From Figure 3a, when no dispersant was added, the particle size of the pigment slurry showed a bimodal distribution in a wide particle size range. Its *D_90_* reached up to 1887 nm with a *I* value as high as 6.55. Besides, the fluidity of the slurry obtained in this case was very poor, which made it very easy to flocculate and quickly precipitate. However, after PAI or PAIN2 was added, the *D_90_* of the pigment slurry was significantly decreased to 293 and 83 nm, respectively. But the particle size of the slurry using PAI as a dispersant still presented an obvious bimodal distribution, which may be because PAI interacted with the CoAl_2_O_4_ particles mainly through a relatively weaker hydrogen bond. After repeated collisions and shear between CoAl_2_O_4_ particles and zirconium dioxide balls, the PAI dispersant would fall off the CoAl_2_O_4_ particle surface, so its grinding effect decreased. PAIN2 could effectively control the particle size distribution of the pigment slurry, which interacted with the CoAl_2_O_4_ pigment particles mainly through the pyrrolidone ligands involved, and therefore can firmly adsorb on the particle surface, playing a good grinding effect. Meanwhile, the carboxyl groups in PAIN2 could provide enough negative charges for CoAl_2_O_4_ particles to generate electrostatic repulsion. To sum up, PAIN2 was favorable for the ultrafine grinding and dispersion stability of the CoAl_2_O_4_ slurry.

Figure 3b displays the particle size distribution of CoAl_2_O_4_ slurry after ultrafine grinding using PAIN1, PAIN2, and PAIN3 as dispersants. It was found that the grinding effect of PAIN2 on the CoAl_2_O_4_ particles was better than that of PAIN1 and PAIN3. The obtained CoAl_2_O_4_ slurry had the smallest *D_90_* of 83 nm and the narrowest particle size distribution (an *I* value as low as 0.42). PAIN1 had a too low pyrrolidone ligand content to produce enough adsorption strength and wettability on the pigment particle surface. So its grinding effect was not very good, and the *D_90_* of the obtained slurry using PAIN1 as a dispersant was too large. However, the pyrrolidone ligand content in PAIN3 was too high. The unadsorbed PAIN3 was dispersed in the solution, causing an increase in the solution viscosity and thus a decreased grinding effect. So the best dispersant was determined to be PAIN2.

Next, the effects of dispersant dosage were discussed. The pigment content and the grinding time were still separately fixed at 20 wt% and 480 min, respectively. Using PAIN2 as a dispersant, the *D_90_* of CoAl_2_O_4_ slurry at different PAIN2 dosages was determined, and the result is given in Figure 3c. As can be seen from Figure 3c, when the PAIN2 dosage was 10 wt%, the grinding effect was the best. The obtained CoAl_2_O_4_ slurry had the smallest particle size and narrowest particle size distribution. When the PAIN2 dosage was between 1 wt% and 5 wt%, the additive amount of dispersant was insufficient, and it was difficult to completely cover the pigment particle surface, leading to poor surface wettability. As the PAIN2 dosage was increased to 15 wt%, the dispersant amount was too much. The superabundant carbon chains were absorbed on the particle surface to entangle each other, which would affect the grinding effect. Meanwhile, the unadsorbed dispersant may cause the solution viscosity to increase, resulting in lower grinding efficiency. Therefore, under this PAIN2 dosage, the grinding effect was poor with a larger particle size and a wider particle size distribution. Ultimately, the optimum dispersant dosage was intended to be 10 wt%.

Finally, the effects of grinding time were considered. In this section, still using PAIN2 as a dispersant, the experiments were conducted to explore the influence of grinding time on the particle size of CoAl_2_O_4_ pigment slurry when the PAIN2 dosage was 10 wt% and the pigment content was 20 wt%, and the results are shown in Figure 3d. It was found that the longer the grinding time, the better the grinding effect. When the grinding time was increased to 480 min, the obtained nano-pigment slurry had good dispersion stability with a *D_90_* as low as 83 nm. As a result, the optimal grinding time was determined to be 480 min.

Based on the above-mentioned measurement result, the optimum wet grinding condition was determined as follows: using PAIN2 as a dispersant, a dispersant dosage of 12 wt% and a grinding time of 480 min. According to this optimum wet grinding condition, the CoAl_2_O_4_ nano-pigment slurry with a pigment content of 20 wt% was prepared to be used for further study.

### 2.3. Characterization and Performance of CoAl_2_O_4_ Nano-Pigment Slurry

**Figure 4:**
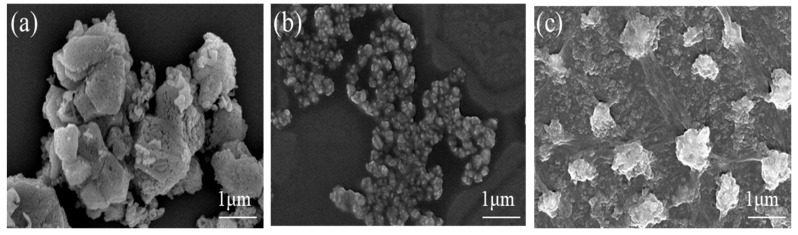
SEM images of CoAl_2_O_4_ raw powder (**a**) and CoAl_2_O_4_ nano-pigment slurry prepared with PAI (**b**) and PAIN2 (**c**).

The SEM images of CoAl_2_O_4_ raw powder as well as CoAl_2_O_4_ nano-pigment slurry prepared with PAI and PAIN2 according to the above-obtained optimum wet grinding technology are displayed in Figure 4. From Figure 4a, the CoAl_2_O_4_ raw powder had a particle size of about 4.0 μm with a poor dispersion. There was an obvious aggregation and stacking phenomenon. From Figure 4b, the particle size of the CoAl_2_O_4_ pigment slurry prepared with PAI as dispersant decreased, but there was still a serious particle aggregation phenomenon. When PAIN2 was used as a dispersant, the agglomeration of the obtained CoAl_2_O_4_ nano-pigment slurry was obviously reduced. Now the CoAl_2_O_4_ particles had a significantly decreased particle size and a narrower particle size distribution (Figure 4c). The above results showed that the grinding effect of PAIN2 was better than that of PAI.

In the CoAl_2_O_4_ nano-pigment slurry, there was a high-frequency Brownian motion between CoAl_2_O_4_ nanoparticles, resulting in a very frequent particle collision. Particularly when the temperature increased, both the frequency and amplitude of Brownian motion were enhanced, and the collision kinetic energy between CoAl_2_O_4_ nanoparticles also rose. As the collision kinetic energy surpassed the repulsive energy barrier, an agglomeration would occur, so the particle sizes would grow. On the basis of Stokes law, as the particle sizes gradually increased, the Brownian motion between nanoparticles would weaken. Now gravity sedimentation played a dominant role, and the system began to precipitate. Therefore, whether the nano-pigment slurry could maintain the particle size unchanged or a little increased after heat storage would directly determine the long-term storage stability.

**Figure 5:**
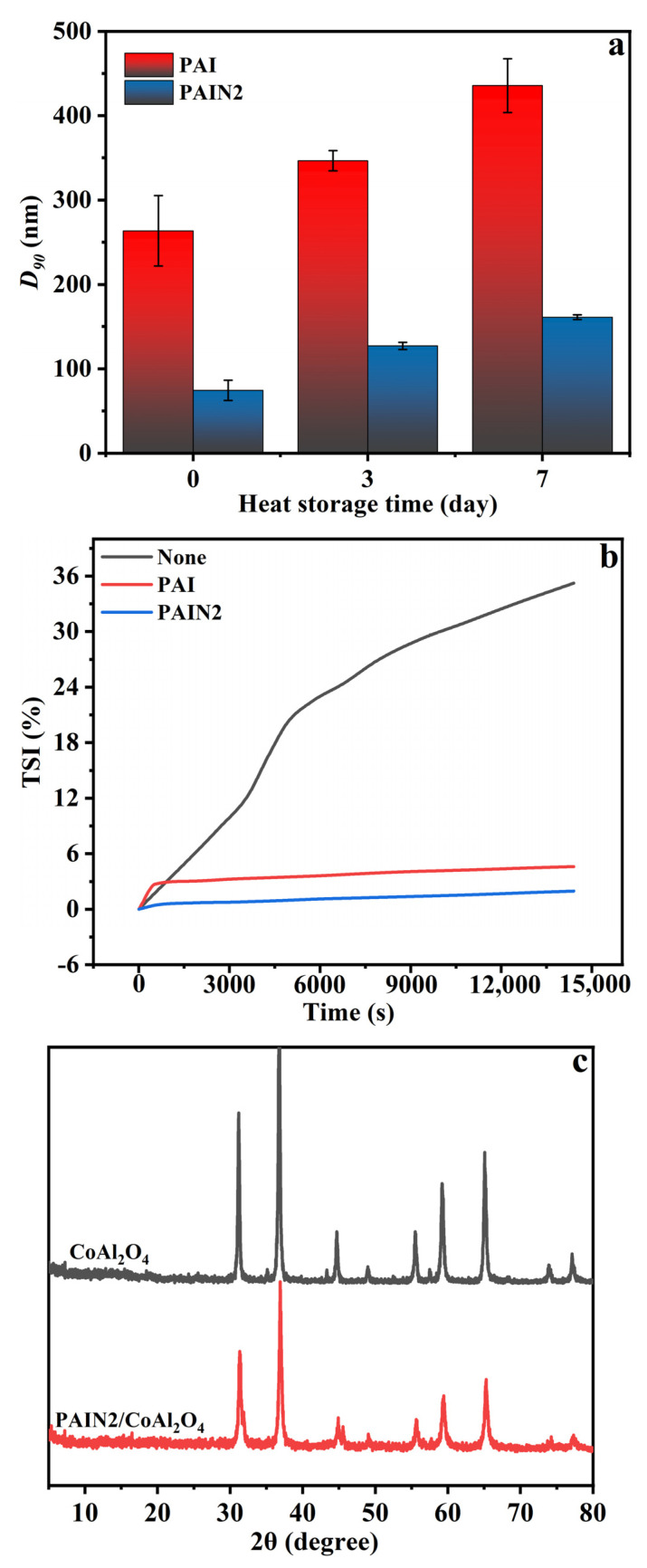
Heat storage stability (**a**) and dispersion stability at room temperature (**b**) of CoAl_2_O_4_ pigment slurry prepared with PAI and PAIN2; XRD spectrogram of CoAl_2_O_4_ raw powder and CoAl_2_O_4_ pigment slurry prepared with PAIN2 (**c**).

In this section, the particle size change of CoAl_2_O_4_ pigment slurry prepared with PAI and PAIN2 before and after heat storage for separately 3 and 7 days under 60 °C was measured to evaluate the heat storage stability, respectively. The results are displayed in Figure 5a. As can be seen from Figure 5a, the *D_90_* of nano-pigment slurry prepared with PAI increased from 293 nm (before heat storage) to 355 nm and 458 nm after heat storage for 3 and 7 days, respectively. When PAIN2 was used as a dispersant, after heat storage for 3 and 7 days, the *D_90_* of the obtained CoAl_2_O_4_ pigment slurry separately increased from 83 nm (before heat storage) to 130 nm and 150 nm (both lower than 200 nm). So the heat storage stability of pigment slurry prepared with PAIN2 was superior to that of pigment slurry prepared with PAI. This was because the adsorption of PAI on the CoAl_2_O_4_ surface was not firm. When the temperature of the pigment slurry increased, the movement of CoAl_2_O_4_ particles intensified, and PAI would fall away from the CoAl_2_O_4_ particle surface, leading to a reduced repulsive energy barrier. So the agglomeration occurred, and the particle size of CoAl_2_O_4_ increased. However, PAIN2 could still adsorb firmly on the CoAl_2_O_4_ particle surface when the temperature rose and the particle movement intensified, providing sufficient electrostatic repulsion and steric hindrance. Thus, the agglomeration between pigment particles was weaker, and the particle size change was smaller. In this case, the CoAl_2_O_4_ particles were still dominated by Brownian motion without precipitation.

Additionally, we also determined the dispersion stability at room temperature of the CoAl_2_O_4_ nano-pigment slurry prepared with PAI and PAIN2, just as shown in Figure 5b. It can be seen that the TSI value of the pigment slurry without any dispersant added reached as high as 35%. When PAI and PAIN2 were used as dispersants, the TSI values were separately 4.6% and 1.9%, indicating improved stability of the pigment slurry after adding dispersant, and the effect of PAIN2 was better than that of PAI. After PAIN2 was added, the particle size of the prepared pigment slurry after grinding was smaller, so the stability was better. Besides, PAIN2 can adsorb on the CoAl_2_O_4_ particles to prevent the collision and sedimentation of nanoparticles through steric hindrance and electrostatic repulsion. Therefore, the nano-pigment slurry prepared with PAIN2 had good stability.

Figure 5c shows the XRD spectrogram of CoAl_2_O_4_ raw powder and CoAl_2_O_4_ pigment slurry prepared with PAIN2. The results showed that the diffraction peak of CoAl_2_O_4_ raw powder was consistent with that of CoAl_2_O_4_ standard card JCPDS 44-0160, indicating that CoAl_2_O_4_ was the main component of CoAl_2_O_4_ pigment. After adding PAIN2 to grind, the diffraction peak and half-peak width of CoAl_2_O_4_ had no obvious changes compared with those of the original CoAl_2_O_4_ pigment, indicating that the crystallinity of pigment particles had no significant changes after grinding. Besides, no new phase was detected in the XRD spectrogram, indicating that the water-based grinding method had the unique advantage of maintaining phase structure characteristics.

### 2.4. Research on the Dispersion Stability Mechanism of PAIN on CoAl_2_O_4_ Nano-Pigment Slurry

In order to further investigate the influence of dispersant on the dispersion stability of CoAl_2_O_4_ particles in the water system, 2 mL of nano-pigment slurry was added to the water (30 mL) to make the pigment particles disperse completely. Afterwards, the Stokes formula and Einstein diffusion law were applied to study the settling factors of CoAl_2_O_4_ particles in water. Specifically, we first calculated the *D* value of CoAl_2_O_4_ in 1 s according to the following Equations (1) and (2) (Stokes formula) [33]:

(1) $$V=\frac{a^{2}\rho g}{18\eta }$$

(2) D=V∗t

where *a* represented the *D_90_* of CoAl_2_O_4_ particles, nm; *V* represented the settling velocity, nm/s; *ŋ* represented the viscosity of dispersing medium, mPa·s; *Δρ* represented the density difference between CoAl_2_O_4_ (4.0 × 10^−3^ kg/cm^3^) and dispersing medium; *g* represented the gravitational acceleration, 9.8 m/s^2^. Since the PAIN concentration in the suspension was very low, its effects were negligible. Assuming that all the suspensions had the same dispersion medium viscosity and density, separately, they were 1 mPa·s, and 1.0 × 10^−3^ kg/cm^3^.

Then, the *X* value of CoAl_2_O_4_ within 1 s was calculated according to the following Equation (3) (Einstein diffusion law) [33]:

(3) X=(RTt3Nπηa)12

where *T* represented the temperature, K; *N* was the Avogadro’s constant, 6.02 × 10^23^; *R* represented the molar gas constant, J/mol·K; *t* represented the diffusion time, s;

Table 2 gives the *D* and *X* values of CoAl_2_O_4_ pigment slurry before and after adding PAI and PAIN2 as dispersants, respectively. From Table 2, the size of the CoAl_2_O_4_ pigment slurry prepared without any dispersant was at a micron level. Meanwhile, its *D* and *X* values were separately 5428 and 481 nm, with *D* much higher than *X*. Therefore, the particle movement in this system was mainly gravity-induced sedimentation, where precipitation was easy to occur. The particle size of CoAl_2_O_4_ pigment slurry prepared with PAI and PAIN2 was at the nanoscale, and the *D* value was separately decreased to 131 and 11 nm, and the *X* value was separately increased to 1221 and 2295 nm, with *X* much higher than *D* in both two cases. So the particles in these two systems were both dominated by Brownian motion, and the sedimentation motion was almost negligible. After adding dispersant to the grind, the particle size was smaller, so the *D* value caused by gravity sharply decreased. Now the particle movement in the slurry was more affected by Brownian diffusion. Because Brownian motion had the characteristics of non-directionality, the particle movement in the nano-scale CoAl_2_O_4_ slurry was also non-directional. The particles would not be easy to sediment, and the dispersion stability of the system was good. As a result, enhancing the dispersion stability by adding dispersant to obtain nano-scale CoAl_2_O_4_ pigment slurry was effective, and generally, the smaller the pigment particle size in the slurry, the higher the stability of the system.

**Figure 6:**
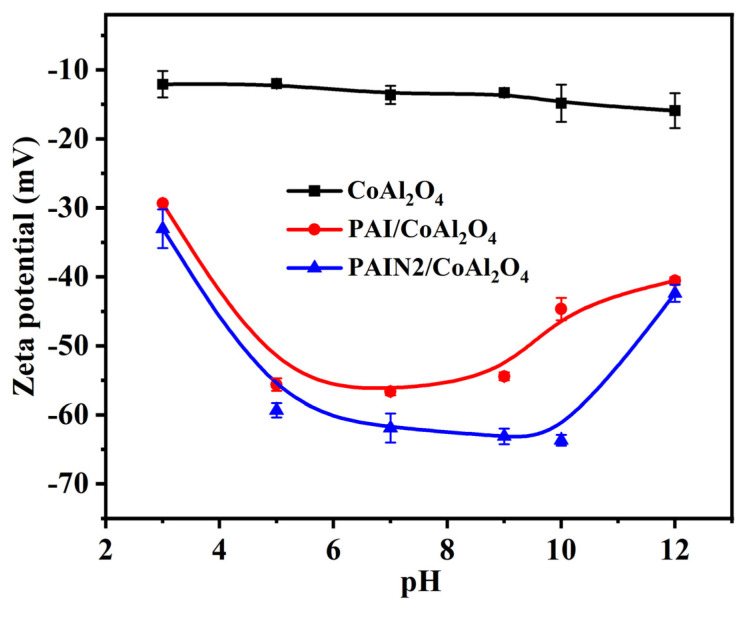
Zeta potential of CoAl_2_O_4_ pigment slurry before and after adding PAI and PAIN2, respectively.

To further elucidate the dispersion mechanism of dispersant, the zeta potential of CoAl_2_O_4_ pigment slurry before and after separately adding PAI and PAIN2 was measured and exhibited in Figure 6. It was found from Figure 6 that the zeta potential of CoAl_2_O_4_ slurry without dispersant was approximately −10 mV, which cannot meet the required potential for particles to maintain a stable dispersion in water. The zeta potential-changing tendency of the CoAl_2_O_4_ slurry prepared with PAI and PAIN2 was consistent with that of the single dispersant aqueous solution, and the lowest potential could be reduced to −56 mV and −64 mV, respectively. This showed that PAI and PAIN2 can provide more negative charges for the particle surface, which is conducive to the electrostatic repulsion between particles so as to maintain the dispersion stability of particles in water. On the basis of DLVO theory, the higher the absolute zeta potential of the particle surface, the stronger the electrostatic repulsion among particles, and the more stable the whole system. So PAIN2 can bring a higher electrostatic repulsive potential energy to CoAl_2_O_4_ particles. This was favorable for the improvement of both the grinding efficiency and stability of the CoAl_2_O_4_ pigment slurry.

**Figure 7:**
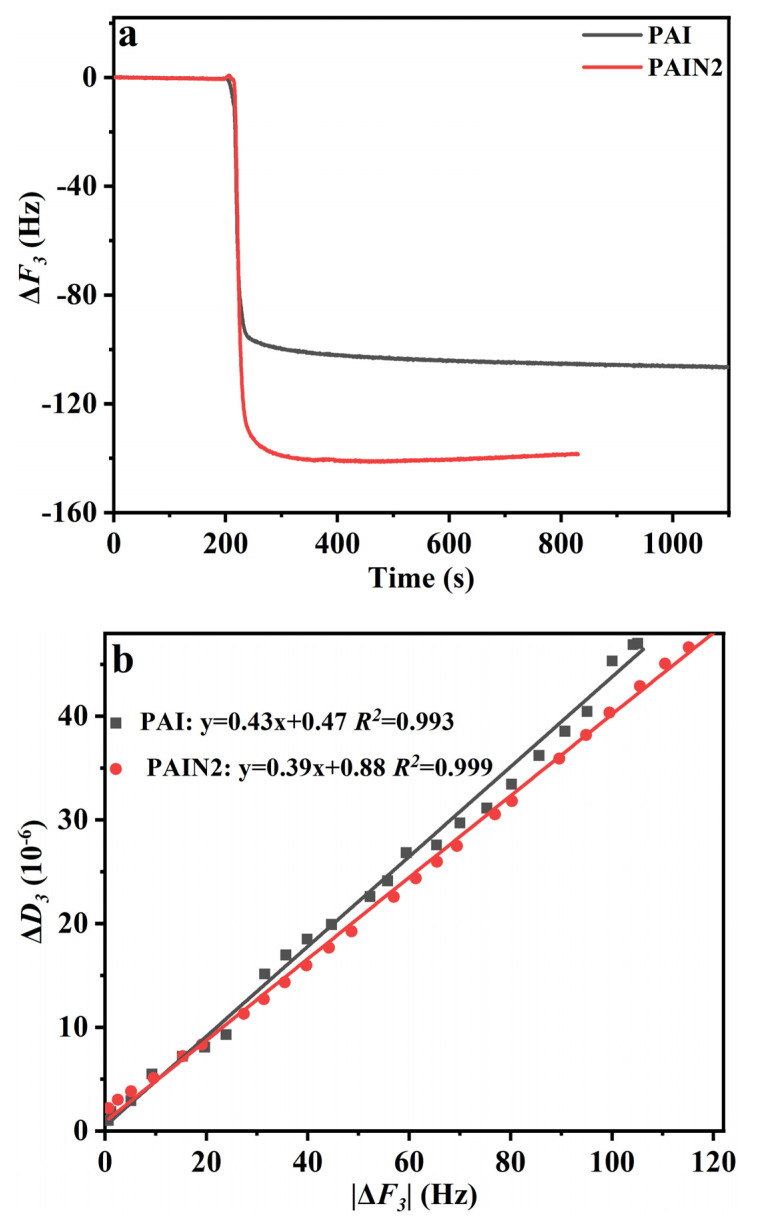
Adsorption characteristics of PAI and PAIN2 on CoAl_2_O_4_ pigment surface (**a**) Adsorption (Δ*F*_3_); (**b**) Δ*D*_3_/|Δ*F*_3_|.

QCM is a highly sensitive mass detection method that can be used to trace the molecular interactions on the nanoscale surface and then to study the adsorption behavior. Among the main testing parameters, the frequency change (Δ*F*) was related to the mass change of the oscillator, so it was often used to represent the adsorption capacity. The dissipation factor change (Δ*D*) was mainly related to the energy loss change of the oscillator system. In order to illustrate the viscoelasticity of the apparent adsorption layer on the vibrator surface, the Δ*D*/|Δ*F*| value was determined. Often, the smaller this value, the better the rigidity of the adsorption layer and the more difficult it is to desorb. In this work, QCM was used to test the adsorption characteristics of PAI and PAIN2 on the CoAl_2_O_4_ pigment surface, respectively. The result was exhibited in Figure 7a and b. From Figure 7a, the absolute Δ*F*_3_ value of PAIN2 was larger than that of PAI, indicating that the adsorption capacity of PAIN2 on the pigment particles was higher than that of PAI. Additionally, the Δ*D*/|Δ*F*| value of PAIN2 was lower than that of PAI, which showed that the formed adsorption layer of PAIN2 on the pigment surface had good viscoelasticity, which was rigid and not easy to fall off (Figure 7b). The analysis indicated that PAI only relied on carboxyl groups to adsorb on the CoAl_2_O_4_ surface, so the adsorption interaction was weaker, and PAI was easy to desorb. However, PAIN2 not only contained carboxyl groups, but also pyrrolidone ligands, which can coordinate with Co-O on the CoAl_2_O_4_ surface. Therefore, the adsorption of PAIN2 on CoAl_2_O_4_ was firm, and the adsorption capacity was large.

**Figure 8:**
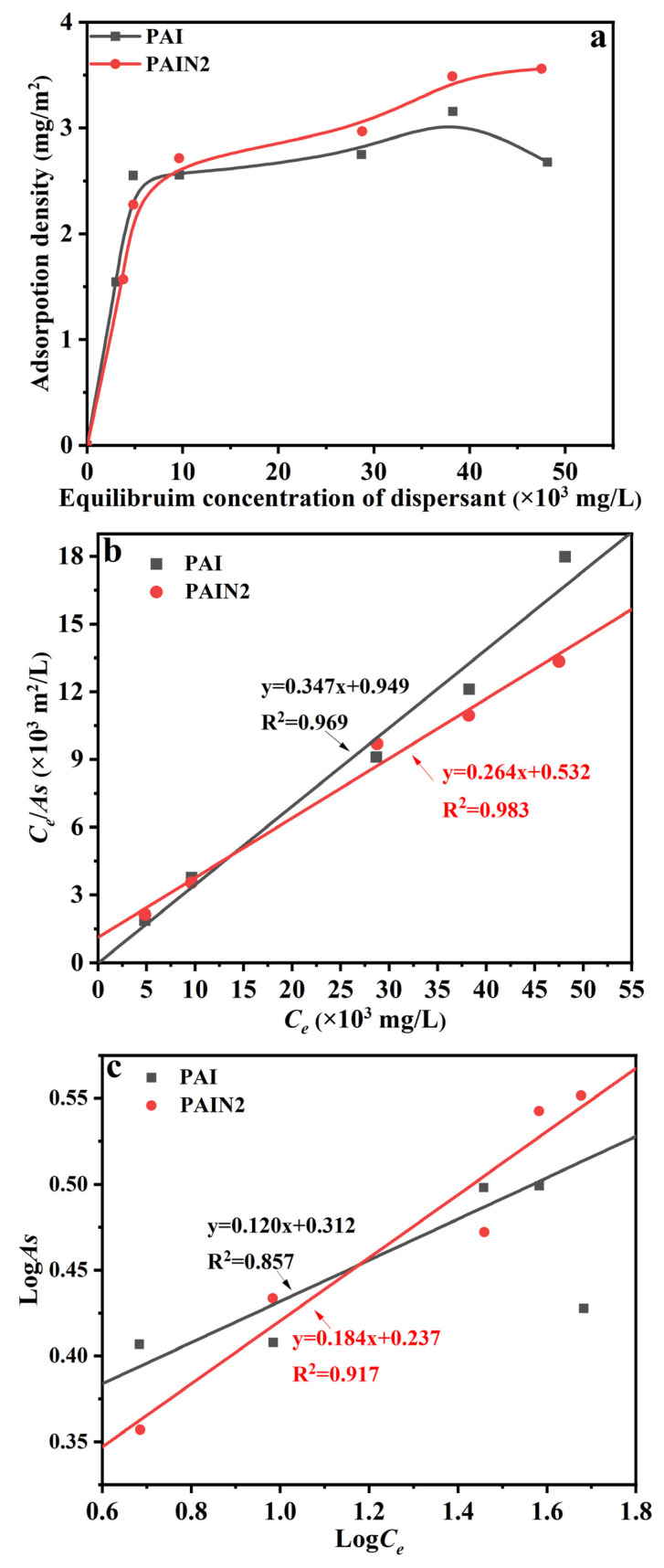
Adsorption isotherm of PAI and PAIN2 on CoAl_2_O_4_ (**a**); fitting results of Langmuir (**b**) and Freundlich (**c**) adsorption models.

According to the DLVO theory, when the dispersant had excellent adsorption performance, its large adsorbability would cause a larger steric hindrance or electrostatic repulsion, making the pigment particles more stable [4]. Therefore, in this section, the adsorption density of PAI and PAIN2 on CoAl_2_O_4_ was determined. Figure 8a displays the adsorption isotherm of PAI and PAIN2 on CoAl_2_O_4_. From Figure 8a, the adsorption densities of PAI and PAIN2 both significantly rose with the increase in the equilibrium concentration of dispersants in solutions and gradually reached a platform. The saturated adsorption density of PAIN2 was approximately 3.5 mg/m^2^, which was significantly larger than that of PAI (2.5–2.8 mg/m^2^). PAI mainly depended on carboxyl groups to adsorb on the CoAl_2_O_4_ surface, so the interaction was relatively weaker. However, the carboxyl groups and pyrrolidone ligands in PAIN2 can both interact with CoAl_2_O_4_, so the adsorption interaction was stronger and the adsorption capacity was larger, which would bring greater electrostatic repulsion and steric hindrance to the pigment particle surface, so that the CoAl_2_O_4_ particles had good dispersion stability in water. Therefore, PAIN2 had an excellent dispersion stabilization effect on the CoAl_2_O_4_ slurry.

In order to further illustrate the adsorption behavior of dispersant on the pigment surfaces as well as quantitatively calculate the saturation adsorption density, Freundlich and Langmuir isothermal adsorption models (the detailed information with regard to these two fitting models is given in the Supplementary Materials) [34] were utilized to fit the adsorption isotherms of PAI and PAIN2 in Figure 8a. The fitting results are exhibited in Figure 8b,c and Table 3. It was found that the linearity of the Langmuir fitting curve was better, which showed both PAI and PAIN2 had a single molecular adsorption layer on the CoAl_2_O_4_ pigment surface. According to the intercept and slope of the Langmuir fitting line, the saturated adsorption density (*As*) of PAI and PAIN2 on the CoAl_2_O_4_ surface can be calculated separately to be 2.9 and 3.8 mg/m^2^.

Then, the *R_A_* value corresponding to the occupied area by each adsorbed dispersant molecule on the pigment surface was calculated from the saturation adsorption density (*C_m_*) according to the following Equation (4) [33]:

(4) $$C_{m}=\frac{S_{A}M_{n}}{\pi {\left(R_{A}\right)}^{2}N}$$

where *M_n_* represented the molecular weight of dispersants; *S_A_* represented the specific surface area of pigment; and *N* represented the Avogadro’s constant, 6.02 × 10^23^.

The result indicated that the *R_A_* values of PAI and PAIN2 separately were 6.1 and 5.4 nm, showing that the occupied area by each PAI adsorbed on the CoAl_2_O_4_ surfaces was larger than that by each PAIN2. The reasons were inferred as follows: PAI was adsorbed on the CoAl_2_O_4_ surface mainly through the carboxyl groups, and the molecules laid flat on the particle surface. PAIN2 mainly relied on pyrrolidone ligands to adsorb on the CoAl_2_O_4_ surface. The adsorption layer was more compact with a three-dimensional structure, so the occupied area by each PAIN2 molecule adsorbed on the CoAl_2_O_4_ surface was smaller, and the adsorption layer thickness and saturation adsorption capacity were larger, which was favorable for producing a greater steric hindrance to improve the particle stability in the suspension.

**Figure 9:**
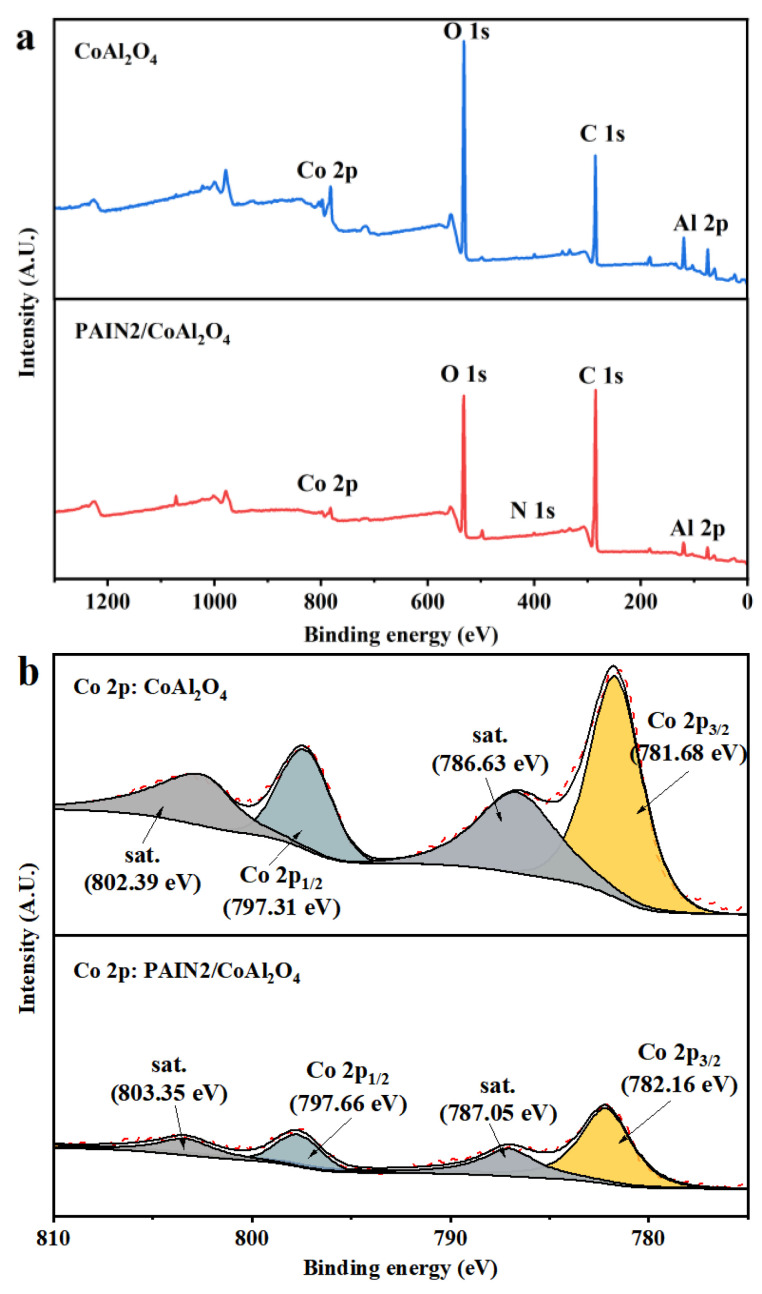

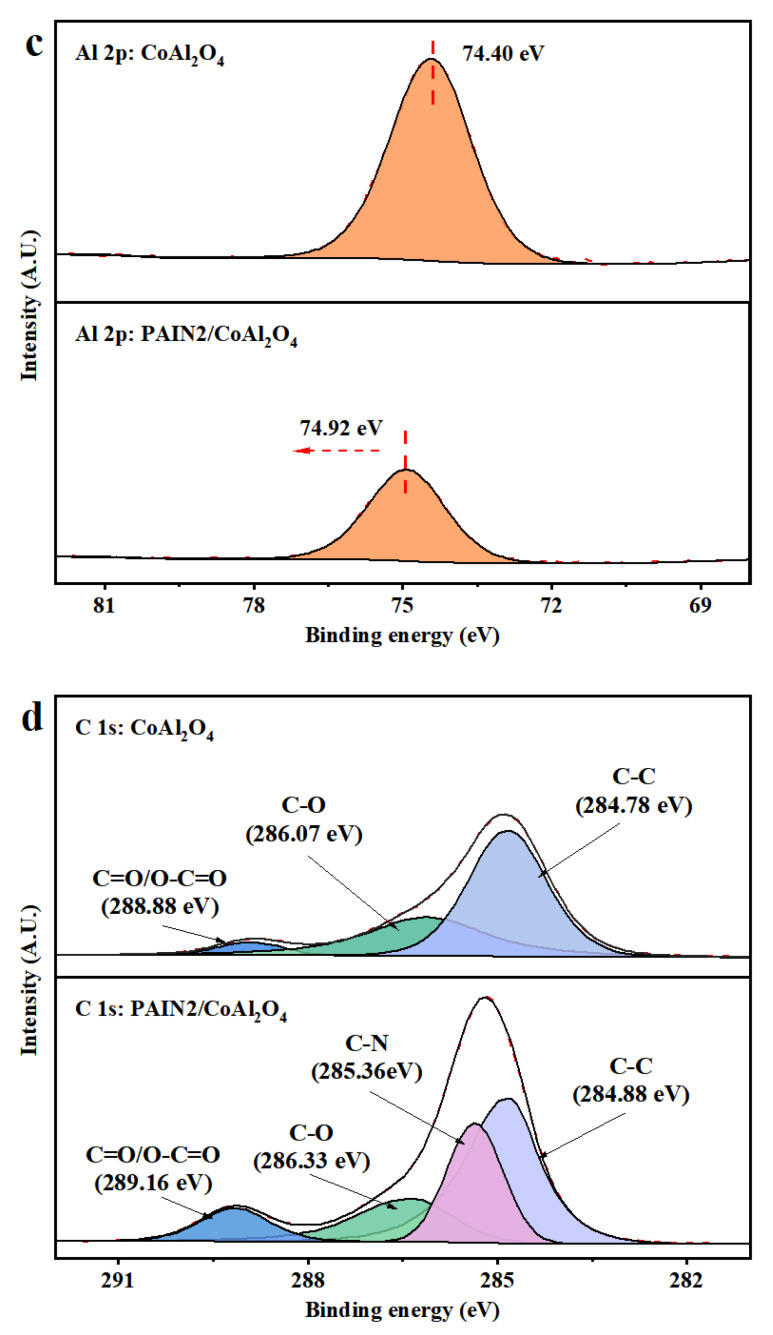
XPS full spectra (**a**), Co 2p spectra (**b**), Al 2p spectra (**c**) and C 1s spectra (**d**) of CoAl_2_O_4_ raw powder and CoAl_2_O_4_ adsorbed by PAIN2.

Next, XPS measurements were performed on CoAl_2_O_4_ raw powder and CoAl_2_O_4_ adsorbed by PAIN2 to explore the adsorption mechanism. The results are given in Figure 9. It can be seen that after PAIN2 was adsorbed on the CoAl_2_O_4_ surface, all the peak intensity and element contents on the CoAl_2_O_4_ full spectra (Figure 9a) obviously changed, and a signal peak appeared at 400.10 eV due to the action of N 1s photoelectrons, indicating that PAIN2 containing N atoms was successfully adsorbed on CoAl_2_O_4_. From the XPS spectra of Co 2p (Figure 9b), it can be clearly seen that there were four peaks at 781.68, 786.63, 797.31, and 802.39 eV, which were the satellite peaks of Co 2p3/2, Co 2p3/2, Co 2p1/2, and Co 2p1/2, respectively. After absorbing PAIN2 on CoAl_2_O_4_, the binding energy of Co 2p separately changed from 781.68 to 782.16 eV, 786.63 to 787.05 eV, 797.31 to 797.66 eV, and 802.39 to 803.35 eV, and the binding energy of Al 2p changed from 74.40 to 74.92 eV (Figure 9c), indicating a strong interaction between PAIN2 and Al/Co [35]. Compared with the three peaks of 284.78, 286.07, and 288.88 eV corresponding to C-C or C-H, C-O, C=O, or O-C=O before adsorption (Figure 9d), due to the adsorption of PAIN2, these three binding energies (284.88, 286.33, and 289.16 eV) all significantly changed after adsorption, and a new C-N peak appeared at 285.36 eV. The above phenomenon may be caused by the interaction of CoAl_2_O_4_ with carboxyl groups and pyrrolidone ligands in PAIN2 [36,37].

**Figure 10:**
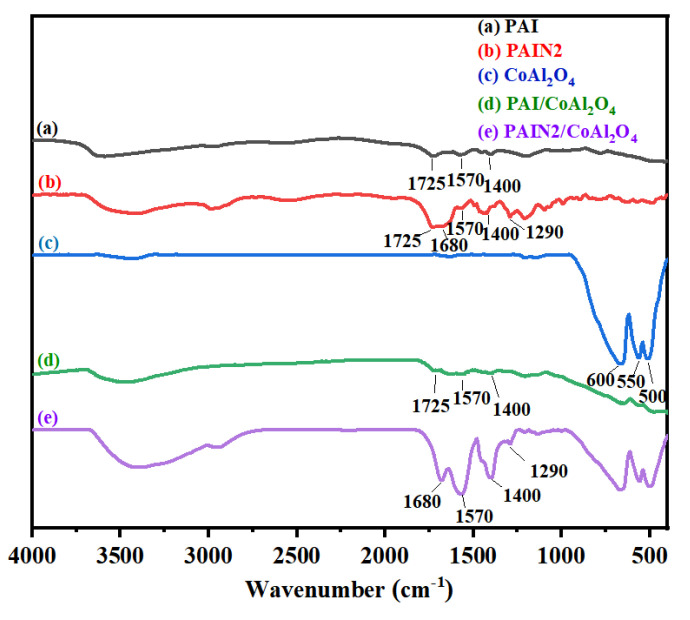
FT–IR spectra of CoAl_2_O_4_ raw powder, CoAl_2_O_4_ powder adsorbed by PAI and PAIN2, and the individual PAI and PAIN2.

Afterwards, we further determined the FT–IR spectra of CoAl_2_O_4_ raw powder, CoAl_2_O_4_ powder adsorbed by PAI and PAIN2, and the individual PAI and PAIN2 to reveal the interaction between dispersants and CoAl_2_O_4_, seeing Figure 10. As can be seen, for CoAl_2_O_4_ particles (spectra a), the peak at 660 cm^−1^ corresponded to the stretching vibration of Al-O, and those at 550 cm^−1^ and 500 cm^−1^ were assigned to the stretching vibration of Co-O. When PAI was adsorbed on CoAl_2_O_4_ (spectra d), the O-H stretching vibration peak appeared at 3430 cm^−1^, and the -COO^−^ characteristic peaks appeared at 1725 cm^−1^, 1570 cm^−1^ and 1400 cm^−1^, respectively. This showed that PAI was adsorbed on CoAl_2_O_4_ mainly by carboxyl groups. When PAIN2 was adsorbed on CoAl_2_O_4_ (spectra e), a new peak appeared at 3430 cm^−1^, which was attributed to the stretching vibration of the overlapping O-H and N-H. Meanwhile, the characteristic peaks of -COO^−^ at 1570 cm^−1^ and 1400 cm^−1^, and the stretching vibration peaks of -C=O and -C-N at 1680 cm^−1^ and 1290 cm^−1^ in the pyrrolidone ligands in PAIN2 also appeared. These results indicated that, besides the role of carboxyl groups, the adsorption of PAIN on CoAl_2_O_4_ particles mainly relied on the coordination adsorption of pyrrolidone ligands. There was a strong coordination interaction between the pyrrolidone ligands in PAIN2 and Co-O in CoAl_2_O_4_ [4,38], so PAIN2 can be adsorbed on the CoAl_2_O_4_ particle surface firmly, endowing the nano-pigment slurry with excellent storage stability.

**Figure 11:**
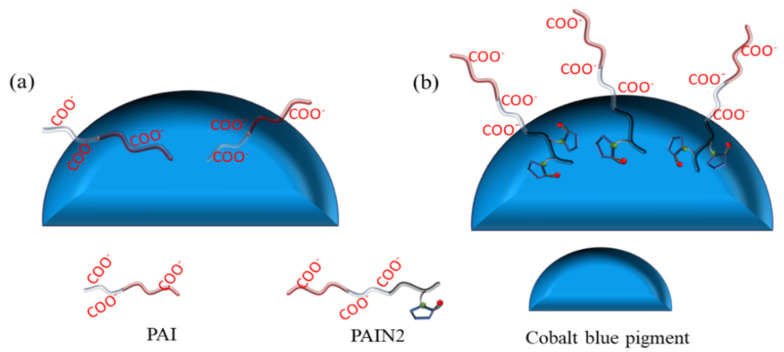
Adsorption models of PAI (**a**) and PAIN2 (**b**) on CoAl_2_O_4_.

Based on the above-mentioned experimental results, the adsorption models of PAI and PAIN2 on CoAl_2_O_4_ were proposed, as shown in Figure 11. PAI depended on carboxyl groups to adsorb on the CoAl_2_O_4_ surface to form a flat adsorption conformation (Figure 11a). PAIN2 relied on its pyrrolidone ligand to form a stable coordination structure with Co-O in CoAl_2_O_4_, so as to adsorb on the CoAl_2_O_4_ surface firmly. The hydrophilic chain segment containing carboxyl groups was oriented towards the solution to enhance the electrostatic repulsion and steric hindrance of the particle surface. The three-dimensional adsorption conformation of PAIN2 (Figure 11b) made the area occupied by a single molecule on the particle surface smaller than PAI, so PAIN2 had a larger adsorption capacity than PAI.

## 3. Materials and Methods

### 3.1. Materials

AA was purchased from Anneji Chemical Co., Ltd. (Shanghai, China). IA and NVP were provided by Macklin Biochemical Technology Co., Ltd. (Shanghai, China). Ammonium persulfate (APS), sodium hydroxide (NaOH), and citric acid were supplied by Aladdin Reagent Co. Ltd. (Shanghai, China). CoAl_2_O_4_ pigment (PB528A) with a specific surface area of 37.6 m^2^/g was purchased from Kelai New Materials Co., Ltd. (Changsha, China).

### 3.2. Synthesis of PAIN

**Figure 12:**
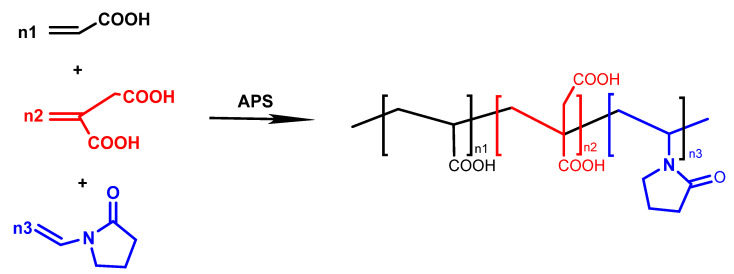
Synthesis route of PAIN.

Using the deionized water as a solvent, three kinds of monomers AA, IA, and NVP were separately added into the four-mouth flask in a certain molar ratio. Then, APS was dropwise added as an initiator through a peristaltic pump within 20–30 min. Next, the flask was heated to a certain temperature and mechanically stirred in a nitrogen atmosphere for a certain time until all monomers reacted completely. When the temperature of the product solution drops to room temperature, its pH value is adjusted to 7–8 with NaOH solutions (20 wt%). Finally, PAIN polymer dispersants with different pyrrolidone ligand contents, named PAIN1, PAIN2, and PAIN3, were obtained by changing the additive amount of NVP. It should be noted that when the additive amount of NVP was 0, the prepared product was named PAI. The synthesis conditions of PAIN were listed in Table 4, and the synthesis route was illustrated in Figure 12.

Afterwards, the above-obtained PAI and PAIN solutions were further purified. Firstly, an excessive amount of ethanol was added, and then it was uniformly agitated for 15 min and put aside for 40 min. Subsequently, the supernatant liquid, including impurities such as the unreacted NVP, was removed. Continuously, the lower precipitation was washed again using ethanol three times. Through evaporating ethanol and freeze drying, the partially obtained PAI and PAIN were used as dispersants to prepare CoAl_2_O_4_ nano-pigment slurry, and others were continuously dealed with a dialysis bag (a cutoff molecular weight of 1000) for five days to remove low-molecular weight impurities such as inorganic salts. Finally, the residual PAI and PAIN solutions were also concentrated through rotary evaporation and freeze drying. The obtained solid PAI and PAIN products were further used for the following structural characterization.

### 3.3. Structural Characterizations of PAIN

The FT–IR spectra measurements of samples were conducted on a Nicolet IS50-Nicolet Continuum FT–IR spectrometer (Thermo Fisher Scientific Co., Waltham, MA, USA). Firstly, the dried solid sample was weighed to mix with KBr at a mass ratio of 1:100 and then ground into fine powders to press into a disk for tests. The testing parameters were set as follows: a scanning wavenumber range of 400–4000 cm^−1^, a scanning time of 32 s, and a resolution of 4 cm^−1^.

The ^1^H NMR spectra of samples were determined by a NMR spectrometer (DRX-400, Bruker, Nehren, Germany). The sample was dissolved in heavy water for measurements under the following conditions: a pulse angle of 60°, a pulse time of 6 s, and a scanning number of 32.

The elemental analysis measurements were carried out on a Vario EL Cube elemental analyzer (Elementar, Langenselbold, Germany). The sample was first dried and ground into powder, and then an appropriate amount of powder was weighed to determine the carbon, hydrogen, and nitrogen contents. Based on this obtained nitrogen element content, the pyrrolidone ligand content in PAIN can be calculated according to the following Equation (5) [4]:

(5) $$\mathrm{Pyrrolidone}\;\mathrm{ligand}\;\mathrm{content}\;(\mathrm{mmol}/g)=\frac{N\%}{14\times 100}\times 1000$$

The molecular weight and distribution of PAI and PAIN were tested through the aqueous water 1515 GPC. The mobile phase was NaNO_3_ solutions with a concentration of 0.1 mol/L and a pH value of 8–9 under a flow rate of 0.5 mL/min. Before measurements, the sample was prepared into 1 mg/mL of solutions, followed by being filtered using a 0.22 μm Millipore filter membrane (Danvers, MA, USA). Afterwards, a certain volume (about 50 μL) of filtered sample was injected into the column for GPC analysis. During tests, sodium polystyrenesulfonate was utilized as a standard, and the sample effluents were monitored by a UV detector (Waters 2487, Waters Corp., Milford, MA, USA).

### 3.4. Preparations of CoAl_2_O_4_ Nano-Pigment Slurry [4]

Firstly, PAIN was dissolved into the deionized water, and its pH value was adjusted to 7–8 using NaOH or citric acid, followed by being mixed with CoAl_2_O_4_ pigment and zirconium beads with a diameter of 0.3 mm (grinding materials) into a YXQM-2L ball mill (Miqi Instrument Co. Ltd., Changsha, China) in accordance with the formula shown in Table S1. Finally, the CoAl_2_O_4_ nano-pigment slurry with a pigment content of 20 wt% was obtained after grinding at a milling speed of 400 rpm.

### 3.5. Performance Characterizations of CoAl_2_O_4_ Nano-Pigment Slurry

The particle size and distribution of CoAl_2_O_4_ pigment slurry were measured through a BetterSize 2600 laser particle analyzer (Dandong Baite Instrument Co. Ltd., Liaoning, China). Taking pure water as a background, the water circulation and ultrasound were turned on. Then, the pigment slurry was dropwise added into the sample pool (controlling the shading rate within 3%) for tests. Each sample was measured at least three times parallelly, and finally the average value was utilized. The particle size distribution slope (uniformity coefficient) was represented by the defect factor (*I*), which can be calculated according to the following Equation (6) [39]:

(6) $$I=\frac{D_{90}-D_{10}}{2D_{50}}$$

where *D_10_*, *D_50_*, and *D_90_* were the corresponding particle sizes when the cumulative particle size distribution number of a sample separately reached 10%, 50%, and 90%. A smaller *I* value generally means a narrower particle size distribution.

Next, the CoAl_2_O_4_ pigment slurry was placed into a drying oven at 60 ± 1 °C for 3 and 7 days, respectively. Afterwards, the particle size of the slurry before and after heat storage was also tested using the above-mentioned laser particle size analyzer, following the same procedure to estimate the heat stability.

The dispersing performance of CoAl_2_O_4_ pigment slurry was measured by a Turbiscan Lab dispersion stabilizer (Formulation, French) at room temperature. The CoAl_2_O_4_ nano-pigment slurry prepared with PAI and PAIN was separately diluted to 0.1 wt% with deionized water to ensure that the light could successfully pass through the pigment slurry suspension during tests. Then, 20 mL of diluted sample was taken out and put into a glass sample bottle, followed by being placed into the sample tank of the instrument. The testing parameters were set as follows: a scanning frequency of 30 s/time, a total scanning time of 4 h and a temperature of 25 °C. The dispersing performance of CoAl_2_O_4_ pigment slurry can be described by the turbiscan stability index (TSI) value. In general, the higher the TSI value, the worse the dispersion stability of the pigment slurry.

The microstructure of CoAl_2_O_4_ nano-pigment slurry could be observed through SEM (SU8220, Carl Zeiss Co., Germany). The pigment slurry prepared with PAI or PAIN2 was diluted with deionized water until it was colorless. Subsequently, this diluted sample was dropped onto the silicon wafer. After being naturally dried, this silicon wafer was subjected to a spraying gold treatment for 60 s and then measured by SEM.

The XRD spectrogram measurements of CoAl_2_O_4_ and CoAl_2_O_4_ nano-pigment slurry prepared with PAIN were performed on an X-ray diffractometer (D8 Advance, Bruker Corp., Karlsruhe, Germany). The CoAl_2_O_4_ pigment slurry was first dried and ground into powder, and then tested under the following conditions: using CuKα radiation as a light source at 40 mA and 60 kV, a 2θ scanning range between 5 and 90°, and a scanning speed of 0.1°/s.

### 3.6. Dispersion Stability Mechanism Study of PAIN on CoAl_2_O_4_ Nano-Pigment Slurry

#### 3.6.1. QCM Measurements

Firstly, 2 wt% of polydiallyl dimethyl ammonium chloride (PDAC) polycationic aqueous solutions were prepared and spin-coated on the surface of a cut sensor crystal (5 MHz-AT) with Au coatings by a spin coater (AC200-SE, Jiangyin Jiatu Technology Co. Ltd., Danyang, Jiangsu, China). Subsequently, 2 wt% of CoAl_2_O_4_ suspension was prepared with deionized water as the dispersion medium and again spin-coated on the above-cut sensor crystal with Au coatings modified with PDAC. Then, 0.5 g/L of PAI and PAIN2 solutions were prepared for use. The program of the spin coater was set below: The spin-coating was first performed at 500 rpm for 30 s, and then at 2000 rpm for 60 s. Finally, the speed was adjusted to 800 rpm for 30 s.

Next, the Q-Sense E1 QCM (Q-Sense AB Corp., Stockholm, Sweden) was utilized to investigate the adsorption behavior of PAI and PAIN2 on the CoAl_2_O_4_ pigment surface at 25 °C. To be specific, the cell was first rinsed with distilled water to establish a stable baseline. Afterwards, the distilled water was replaced by PAI or PAIN2 solutions, and at the same time, the data were recorded. During the whole process, the flow rate of the mobile phase was always kept at 0.15 mL/min.

#### 3.6.2. Adsorption Capacity Measurements of PAIN on CoAl_2_O_4_

The CoAl_2_O_4_ pigment slurry prepared with PAI or PAIN2 was centrifuged for 30 min at 10,000 r/min, and then the supernatant was removed. Afterwards, the obtained lower precipitate was washed with distilled water and subsequently dried in a vacuum drying oven for 12 h. Next, a thermogravimetric measurement through a TG209F3 thermogravimetric analyzer (NETZSCH, Corp., Selb, Germany) was performed to determine the saturation adsorption capacity (*m*, mg) of dispersant on the CoAl_2_O_4_ particles according to the weight loss in the heating process. The adsorption density (*As*) of dispersant on the CoAl_2_O_4_ particles and the equilibrium concentration (*C_e_*) of dispersant in the slurry could be calculated by the following Equation (7) and Equation (8), respectively:

(7) As=mMSBET

where mM was the mass of dispersant adsorbed by the unit mass of CoAl_2_O_4_ particles, mg/g; *S_BET_* was the specific surface area of CoAl_2_O_4_ particles, 37.6 m^2^/g.

(8) $$C_{e}=\frac{m_{0}-m}{V}$$

where *m*_0_ was the total mass of dispersant added, mg; *V* was the suspension volume, L.

#### 3.6.3. Zeta Potential Measurements

The zeta potential was determined on a zeta potential analyzer (BI-90Plus, (Brookhaven Instruments Corp., Holtsville, NY, USA). Each sample was prepared into a solution with a concentration of 1 g/L, and then the pH value was adjusted to 3–12 with citric acid or NaOH solutions (20 wt%) for tests.

#### 3.6.4. XPS Measurements

The XPS spectra of CoAl_2_O_4_ raw powder and CoAl_2_O_4_ adsorbed by PAIN2 were determined by a K-Alpha multifunctional X-ray photoelectron spectrometer (Thermo Fisher Scientific Co., USA). The sample was first dried in a vacuum oven for 12 h. Then, 10 mg of dried sample was weighted for measurements using Al Kα X-ray as an excitation source under an operating voltage of 12 kV, a filament current of 6 ma, and a beam spot of 400 μm.

#### 3.6.5. FT–IR Measurements

The CoAl_2_O_4_ raw powder and CoAl_2_O_4_ pigment slurry prepared with PAI and PAIN2 were dried in a drying oven. Then, their FT–IR spectra were also determined by the Nicolet IS50-Nicolet Continuum FT–IR spectrometer (Thermo Fisher Scientific Co., USA) according to the above method described in Section 2.3.

## 4. Conclusions

PAI and a series of PAIN dispersants with a pyrrolidone ligand content between 1.89 and 3.38 mmol/g were synthesized by the free radical polymerization of NVP with AA and IA. The FT–IR, NMR, EA, and GPC measuring results proved the successful synthesis of PAI and PAIN. Then, the CoAl_2_O_4_ nano-pigment slurry with a pigment content of 20 wt% was prepared by a water-based grinding method, and the optimum grinding conditions were explored and obtained as follows: PAIN2 as a dispersant, a dispersant dosage of 10 wt%, and a grinding time of 480 min. Based on this optimum grinding technology, the prepared CoAl_2_O_4_ nano-pigment slurry had obviously decreased agglomeration. The *D_90_* of this pigment slurry was 82 nm, and separately increased to 130 nm and 150 nm after heat storage for 3 and 7 days (both still lower than 200 nm), showing excellent heat storage stability. Besides, its TSI value was the lowest (1.9%), suggesting good dispersion stability at room temperature. The XRD results indicated that the main crystalline structure of CoAl_2_O_4_ in the pigment slurry was maintained after grinding. Additionally, this prepared nano-pigment slurry by PAIN2 had the largest absolute zeta potential value, which was favorable for the electrostatic repulsion between CoAl_2_O_4_ particles. The QCM characterization and adorption capacity measuring results showed PAIN2 had a larger adsorption capacity on CoAl_2_O_4_, and the formed adsorption layer had a higher rigidity and was not easy to fall off. Besides, each adsorbed PAIN2 molecule occupied a smaller area on the CoAl_2_O_4_ surface. This was because not only PAIN2 could depend on carboxyl groups to adsorb on the CoAl_2_O_4_ surface, but there was also a strong coordination interaction between the pyrrolidone ligands in PAIN2 and Co-O in CoAl_2_O_4_. Further XPS and FT–IR characterizations proved this adsorption mechanism. Therefore, PAIN2 can adsorb on the CoAl_2_O_4_ surface firmly to generate a great electrostatic repulsion and steric hindrance, and this prepared CoAl_2_O_4_ nano-pigment slurry by PAIN2 has the potential to maintain long-term dispersion stability. This work developed a new high-performance CoAl_2_O_4_ nano-pigment slurry. In the future, this obtained nano-pigment slurry prepared with PAIN could be used as inkjet printing inks for ceramic surface decoration. A future study on this application would be conducted.

## Figures and Tables

**Table 1:** Elemental analysis, pyrrolidone ligand content, and molecular weight data of PAI and PAIN.

Dispersant	C%	H%	N%	Pyrrolidone Ligand Content (mmol/g)	*M_w_*	*M_n_*	PDI
PAI	44.51	4.23	0	--	43,890	12,590	3.5
PAIN1	43.21	5.53	2.64	1.89	42,830	15,130	2.8
PAIN2	47.78	6.41	4.43	3.16	27,620	13,150	2.1
PAIN3	43.10	7.51	4.73	3.38	14,240	6470	2.2

**Table 2:** *D* and *X* values of CoAl_2_O_4_ pigment slurry before and after adding PAI and PAIN2 as dispersants.

Dispersant	*a*/*D_90_* (nm)	*V* (nm/s)	*D* (nm)	*X* (nm)
None	1887	5428	5428	481
PAI	293	131	131	1221
PAIN2	83	11	11	2295

**Table 3:** Fitted parameters of PAI and PAIN2 obtained from Langmuir and Freundlich adsorption models, respectively.

Dispersant	Langmuir			Freundlich		
	*As* (mg/m^2^)	*K*_1_ (mg/L)	*R* ^2^	*n*	*K_f_*	*R* ^2^
PAI	2.9	2.752	0.969	7.207	2.051	0.857
PAIN2	3.8	2.021	0.983	5.712	1.726	0.917

**Table 4:** Synthesis conditions of PAIN.

Dispersant	Molar Ratio of AA, IA and NVP (n1:n2:n3)	Additive Amount of APS (wt%)	Temperature (°C)	Reaction Time (h)
PAI	1:1:0	3	85	8
PAIN1	1:1:0.5	1	85	6
PAIN2	1:1:1	1	80	6
PAIN3	1:1:2	1	80	6

## Data Availability

Data are contained within the article and Supplementary Materials.

## Supplementary Materials

The following supporting information can be downloaded at: https://www.mdpi.com/article/10.3390/molecules29163940/s1, Detailed information with regard to Freundlich and Langmuir isothermal adsorption models; Table S1: Grinding formula for preparing CoAl_2_O_4_ nano-pigment slurry.
