# Supplementary material for: Preparation of Modified Polycarboxylate by Pyrrolidone for Using as a Dispersant in Cobalt Blue Nano-Pigment Slurry
Source: Molecules. 2024 Aug 21;29(16):3940. doi: 10.3390/molecules29163940 (PMC11357071; doi:10.3390/molecules29163940)
Supplement: Supplementary file 1 [file molecules-29-03940-s001.zip › molecules-3138056-supplementary.pdf]

Supplementary Materials

Preparation of Modified Polycarboxylate by Pyrrolidone for Using as a Dispersant in Cobalt Blue Nano-Pigment Slurry

Qianqian Tang [1], Rong Yang [2], Jinnuo Li [1], Mingsong Zhou [2,*] and Dongjie Yang [2]

[1] Henan Key Laboratory of Function-Oriented Porous Materials, College of Chemistry and Chemical Engineering, Luoyang Normal University, 6 Jiqing Road, Yibin District, Luoyang 471934, China; lhltqq1987@163.com (Q.T.); 17333740259@163.com (J.L.)

[2] State Key Laboratory of Pulp and Paper Engineering, School of Chemistry and Chemical Engineering, South China University of Technology, 381 Wushan Road, Tianhe District, Guangzhou 510640, China; yr662934@163.com (R.Y.); cedjyang@scut.edu.cn (D.Y.)

* Correspondence: mingsongzhou99@163.com; Tel.: +86-20-87114722

Number of Pages: 3
Number of Tables: 1
Detailed information with regard to Freundlich and Langmuir isothermal adsorption models.
Table S1. Grinding formula for preparing $CoAl_2O_4$ nano-pigment slurry.

Detailed information with regard to Freundlich and Langmuir isothermal adsorption models:

The Langmuir adsorption isotherm model assumed that the surface of the adsorbing material was uniform, and

the adsorbed substance was adsorbed on the solid surface in the form of a single molecular layer, and there was no interaction between the adsorbed molecules. The Langmuir adsorption equation was shown as follows:

$$\frac{C_e}{As} = \frac{C_e}{C_m} + \frac{K}{C_m} \quad \text{(S1)}$$

Where As was the adsorption density, mg/m^2; C_e was the equilibrium concentration of dispersant in the solution, mg/L; C_m was the saturation adsorption density, mg/m^2; K was a constant, mg/L.

The Freundlich adsorption isothermal model often took into account of the molecular interaction between the adsorbing material and the adsorbed substance, and was utilized to describe the multilayer adsorption of a substance on the heterogeneous surface. The Freundlich models could be expressed as follows:

$$LogAs = Logk_f + \frac{1}{n} LogC_e \quad \text{(S2)}$$

Where C_e was the equilibrium concentration of dispersant in the solution, mg/L; As was the equilibrium adsorption density, mg/m^2; k_f was a constant which was related to the adsorption capacity, mg/m^2; n was a dimensionless constant, which was related to the heterogeneity degree and adsorption strength of the adsorbing material surface and can be used to judge the adsorption favourability. When $0.1<1/n<1$, the adsorption process was easy. However, when $1/n>1$, the result was just opposite.

Table S1. Grinding formula for preparing $CoAl_2O_4$ nano-pigment slurry.

Components	Materials	Dosage (wt%)
Pigment	$CoAl_2O_4$	20
Dispersant	PAI or PAIN	10
pH regulator	NaOH or citric acid	≈1
Dispersion medium	Deionized water	69
Total		100
